# Extracardiac Tamponade and Hemoperitoneum After Cardiopulmonary Resuscitation: Multimodal Diagnosis Using Point-of-Care Ultrasonography and Transesophageal Echocardiography

**DOI:** 10.7759/cureus.114219

**Published:** 2026-08-09

**Authors:** Klint J Smart, Oluwakemi Tomobi, Anna L Zukowskia, Scott Heller, Jonathan Bond

**Affiliations:** 1 Anesthesiology, West Virginia University School of Medicine, Morgantown, USA

**Keywords:** cardiopulmonary resuscitation, extracardiac tamponade, focused assessment with sonography for trauma, hemodynamic instability, hemoperitoneum, hepatic hematoma, point-of-care ultrasonography, post-cardiac arrest shock, splenic injury, transesophageal echocardiography

## Abstract

Cardiopulmonary resuscitation (CPR) is associated with iatrogenic complications, including thoracic and intra-abdominal injuries. We present two cases of post-resuscitation hemodynamic instability caused by intra-abdominal hemorrhage. In the first case, hemoperitoneum from splenic injury was rapidly identified using point-of-care ultrasonography (POCUS), prompting emergent surgical intervention. In the second case, a hepatic hematoma resulted in extrinsic compression of the right atrium (RA) and right ventricle (RV), producing extracardiac tamponade physiology confirmed with transesophageal echocardiography (TEE).

These cases highlight the importance of early multimodal imaging in patients with unexplained shock following return of spontaneous circulation (ROSC). These complications are relevant to anesthesiologists and intensivists, who are frequently responsible for the perioperative and critical care management of patients following cardiac arrest.

## Introduction

Cardiopulmonary resuscitation (CPR) remains essential for achieving return of spontaneous circulation (ROSC) following cardiac arrest, but is associated with iatrogenic complications. While thoracic injuries such as rib and sternal fractures are well described, intra-abdominal injuries are less commonly recognized and may contribute to significant morbidity. Advanced imaging studies suggest that clinically significant visceral injuries occur more frequently than previously appreciated, with hepatic and splenic injuries among the most important causes of post-resuscitation hemorrhage [1-4].

Solid organ injury following CPR is uncommon but may result in hemorrhagic shock and delayed diagnosis. Recognition of these complications can be particularly challenging because persistent hemodynamic instability after ROSC is often attributed to myocardial dysfunction, vasoplegia, or recurrent ischemia rather than occult intra-abdominal bleeding. Contemporary post-resuscitation care appropriately prioritizes neurologic recovery, coronary reperfusion, and optimization of cardiac function; however, traumatic complications of CPR may receive comparatively less attention despite their potential to cause ongoing hemodynamic instability [1,2,5,6].

Because many post-cardiac arrest patients are too hemodynamically unstable for immediate transport to obtain advanced imaging, bedside ultrasonography may provide the first opportunity to identify occult sources of hemorrhage. POCUS, particularly the extended focused assessment with sonography in trauma (eFAST), enables rapid bedside detection of hemoperitoneum by clinicians in unstable patients, while transesophageal echocardiography (TEE) provides a continuous assessment of cardiac function and can identify uncommon mechanisms of hemodynamic compromise, including extracardiac cardiac compression, when transthoracic imaging is limited [7-9]. In addition to identifying conventional cardiac pathology, TEE can detect extracardiac tamponade, a rare form of obstructive shock caused by external compression of cardiac chambers by adjacent structures, impairing cardiac filling and reducing stroke volume [10].

Although isolated cases of hepatic and splenic injury after CPR have been described, reports showing the complementary use of point-of-care ultrasonography (POCUS) and TEE to diagnose both intra-abdominal hemorrhage and extracardiac tamponade physiology remain limited. We present two cases illustrating how multimodal bedside imaging facilitated the diagnosis of life-threatening intra-abdominal complications following CPR, including a rare case of extracardiac tamponade caused by a hepatic hematoma [3,4,7].

## Case presentation

Case 1

A 50-year-old patient with a history of multiple sclerosis, hypertension, and hyperlipidemia presented following a witnessed cardiac arrest. ROSC was achieved after one cycle of CPR and defibrillation by emergency medical services. Electrocardiography showed ST-segment elevation myocardial infarction, and the patient received aspirin, intravenous heparin, ticagrelor, and tenecteplase prior to transfer for further management. The patient arrived in the intensive care unit hemodynamically stable, on a norepinephrine infusion at a rate of 0.06 mcg/kg/min.

Left heart catheterization revealed diffuse stenosis of the diagonal branch that was not amenable to percutaneous coronary intervention. Following the procedure, the patient developed hemodynamic instability requiring vasopressor and inotropic support (Table 1). Laboratory evaluation demonstrated a significant decline in hemoglobin concentration from 13.9 g/dL to 7.6 g/dL and lactic acidosis (Table 1).

**Table 1:** Key laboratory and hemodynamic findings.

Variable	Results Upon ICU Arrival	At the Time of Deterioration	Units
Hemoglobin	13.9	7.6	g/dL
Lactic acid	2.2	6.4	mmol/L
pH	7.45	7.17	-
Mean Arterial Pressure	72	61	mmHg
Heart Rate	89	115	beats/min
Vasopressors	Norepinephrine 0.06 mcg/kg/min	Epinephrine 0.06 mcg/kg/min, Norepinephrine 0.1 mcg/kg/min	-

**Figure 1:**
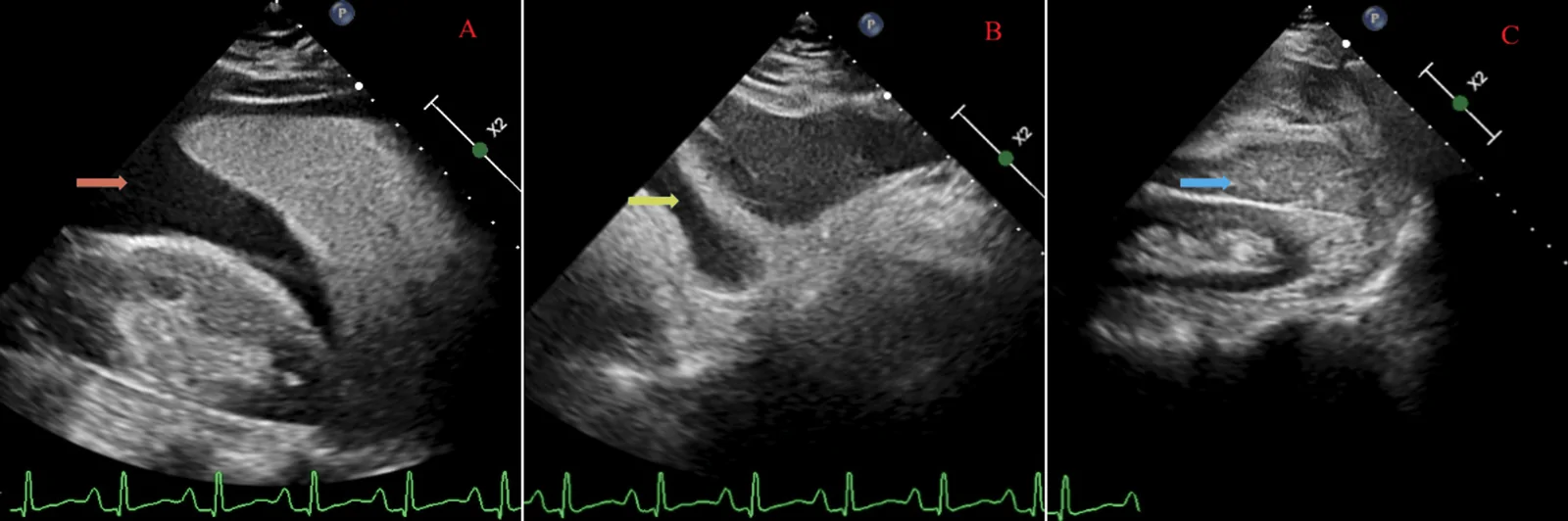
(A) Free fluid within the hepatorenal recess (red arrow). (B) Free fluid within the vesicouterine pouch (yellow arrow). (C) Splenorenal view showing heterogeneous splenic parenchyma, suggesting splenic injury (blue arrow).

POCUS demonstrated mildly reduced left ventricular (LV) function with anterior wall hypokinesis. In addition, an eFAST revealed free intraperitoneal fluid within the hepatorenal recess and pelvis, raising concern for hemoperitoneum and possible splenic injury (Figure 1).

The patient was resuscitated with a total of 9 units of packed red blood cells, 6 units of fresh frozen plasma, and 1 unit of platelets. Once stabilized, computed tomography (CT) confirmed splenic injury with active bleeding. She was taken emergently to the operating room and underwent an exploratory laparotomy with splenectomy. The patient had an uncomplicated postoperative course and was subsequently discharged home.

Case 2

A 58-year-old patient presented with cardiogenic shock following out-of-hospital cardiac arrest due to ventricular fibrillation. ROSC was achieved after multiple defibrillation attempts. The patient arrived at the intensive care unit on the following vasoactive infusions: epinephrine 0.04 mcg/kg/min, norepinephrine 0.08 mcg/kg/min, and vasopressin 0.03 units/min (Table 2). Laboratory values showed elevated creatinine, transaminitis, lactic acidosis, hypoxemia, markedly elevated high-sensitivity troponin I, and ECG findings consistent with a non-ST-segment elevation myocardial infarction (Table 3).

**Table 2:** Hemodynamic findings. IABP: intra-aortic balloon pump

Parameter	Results Upon ICU Arrival	Results After IABP Placed	Results 36 Hours After Arrival	Units
Cardiac Index	1.78	2.9	1.9	L/min/m²
Systemic Vascular Resistance	1,237	692	900	mmHg
Central Venous Pressure	11	6	19	mmHg
Pulmonary Artery Systolic Pressure	43	28	46	mmHg
Pulmonary Artery Diastolic Pressure	24	10	23	mmHg
Mean Pulmonary Artery Pressure	31	14	33	mmHg
Mean Arterial Pressure	68	74	58	mmHg
Heart Rate	111	98	104	beats/min
Vasopressors	Epinephrine 0.04 mcg/kg/min, Norepinephrine 0.08 mcg/kg/min, Vasopressin 0.03 units/min	Dobutamine 2.5 mcg/kg/min, Norepinephrine 0.02 mcg/kg/min	Dobutamine 2.5 mcg/kg/min	-

**Table 3:** Key laboratory findings.

Test	Results Upon ICU Arrival	Results 24 Hours After Arrival	Results 36 Hours After Arrival	Units
Hemoglobin	13.7	11.7	6.8	g/dL
Platelet count	325	152	117	x 10^3^/uL
Blood urea nitrogen	24	32	57	mg/dL
Creatinine	1.59	1.72	2.46	mg/dL
Alanine aminotransferase	1,556	924	1,017	U/L
Aspartate aminotransferase	1,005	727	951	U/L
High sensitivity troponin-I	53,979 ng/L	>60,000	>60,000	ng/L
Lactic acid	8.5 mmol/L	1.2	2.2	mmol/L
PaO₂/FiO₂ ratio	75	252	247	-

Left and right heart catheterization showed severe multivessel coronary artery disease, elevated LV end-diastolic pressure, and a low cardiac index (Table 2). An intra-aortic balloon pump was placed for hemodynamic support, with improvement in the patient's cardiac index and left-sided filling pressures (Table 2). Approximately 36 hours after arrival to the intensive care unit, the patient developed worsening hemodynamic instability, with declining hemoglobin from 11.7 g/dL to 6.8 g/dL (Table 3). Cardiac tamponade was suspected given a low cardiac index, significant pulse pressure variation on arterial monitoring, along with elevation and equalization of pulmonary artery diastolic pressure and central venous pressure (Table 2).

**Figure 2:**
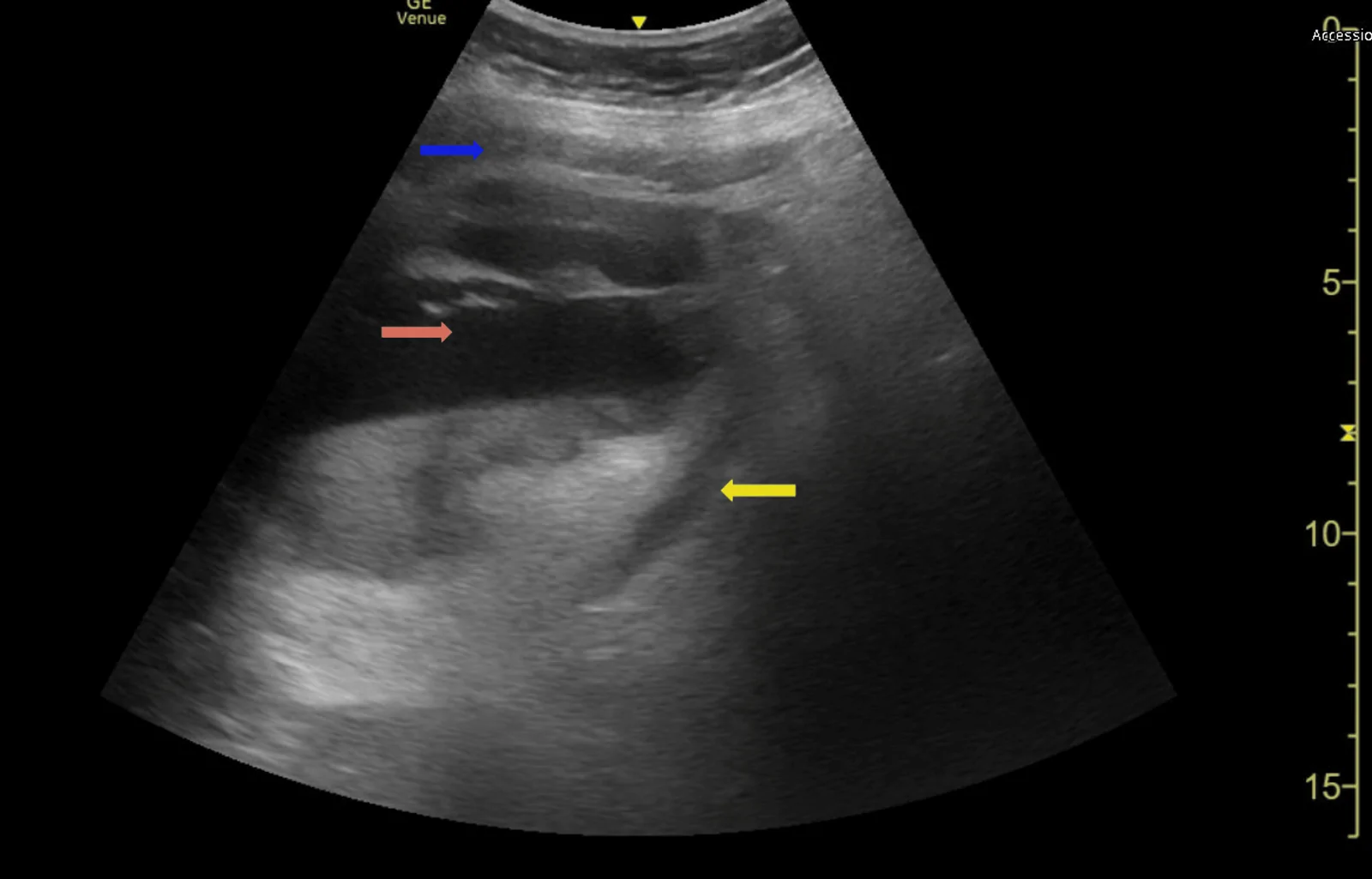
Abdominal POCUS demonstrating a subcapsular hepatic hematoma (blue arrow) and an intraparenchymal hepatic hematoma (red arrow), with free intraperitoneal fluid along the inferior border of the liver (yellow arrow). POCUS: point-of-care ultrasonography

A large subcapsular and intraparenchymal liver hematoma with free fluid around the liver (Figure 2) was seen on eFAST.

**Figure 3:**
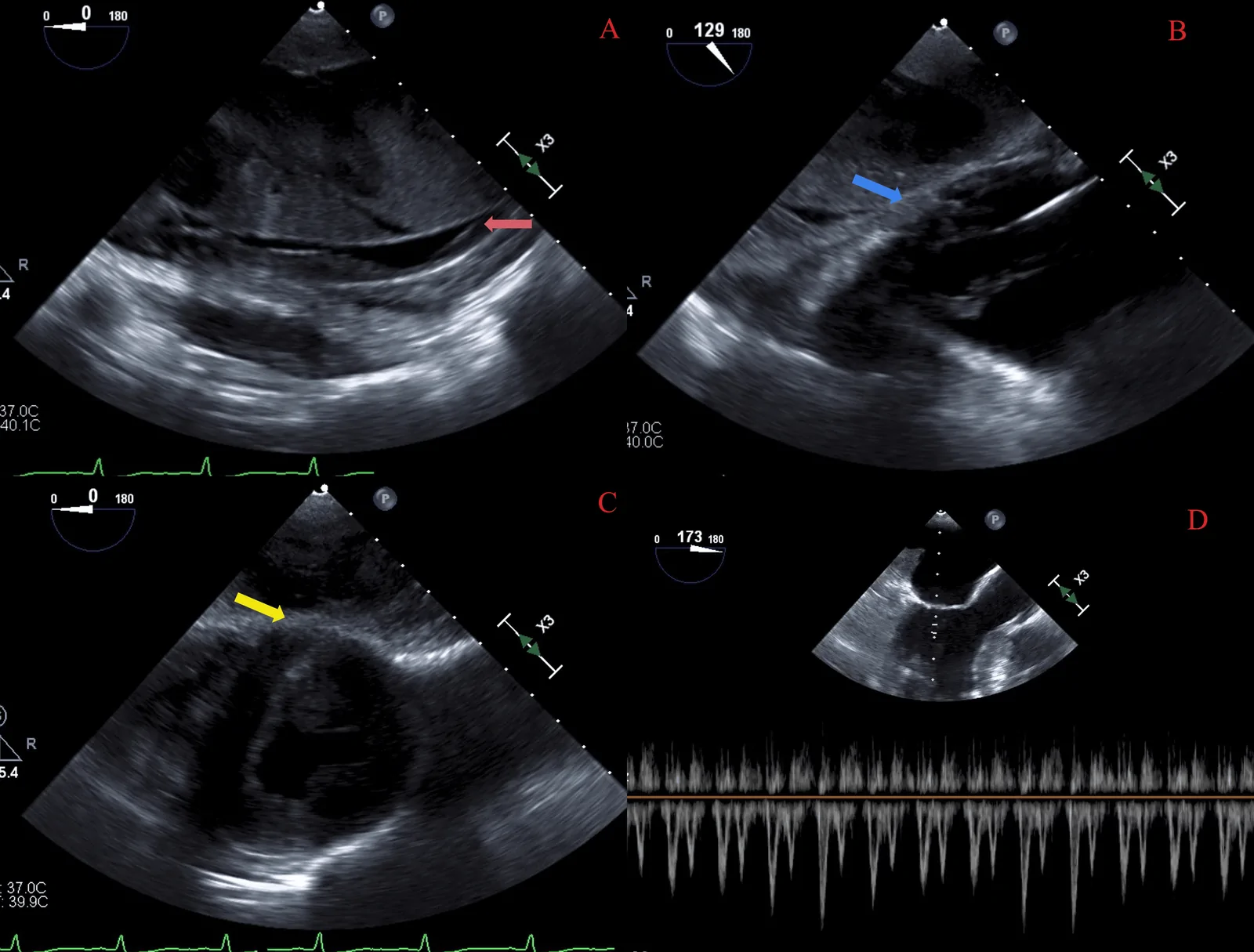
Transesophageal echocardiography. (A) Transgastric view demonstrating hepatic laceration with a subcapsular hematoma (red arrow) causing external compression of adjacent cardiac structures. (B) Transgastric RV inflow view showing extrinsic compression of the RV by the liver (blue arrow). (C) Transgastric mid-papillary short-axis view demonstrating the liver abutting the inferior borders of the RV and LV (yellow arrow), with associated interventricular septal flattening during diastole. (D) Pulsed-wave Doppler of mitral inflow demonstrating significant respiratory variation in E-wave velocity (43%), consistent with tamponade physiology. RV: Right Ventricle; LV: Left Ventricle; E-wave: Early Rapid Filling Wave

**Figure 4:**
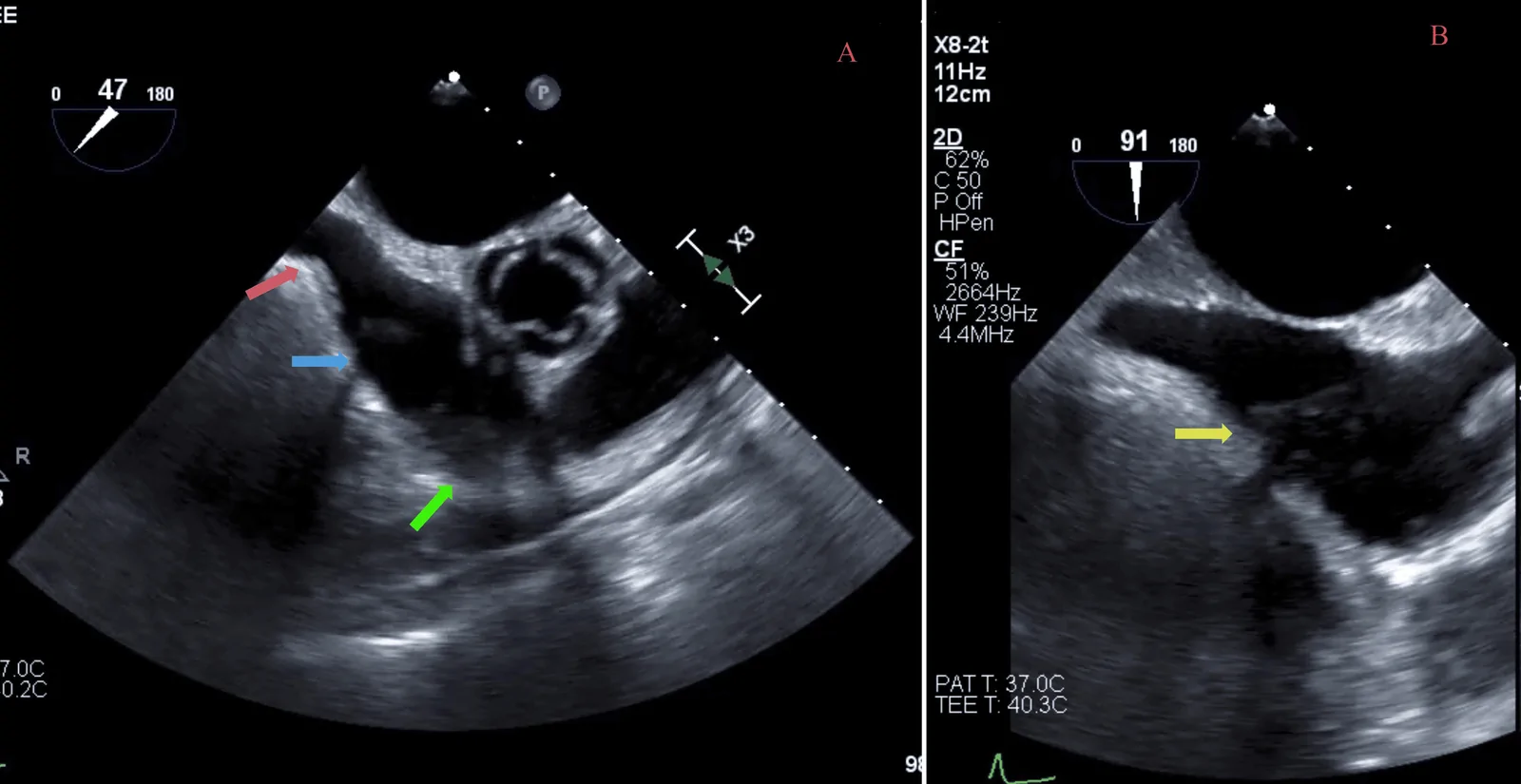
Transesophageal echocardiography. (A) Midesophageal RV inflow-outflow view demonstrating extrinsic compression of the RA-inferior vena cava junction (red arrow), RA (blue arrow), and RV free wall (green arrow). A small pericardial effusion is also present along the RV free wall. (B) Midesophageal bicaval view demonstrating RA compression (yellow arrow). RV: Right Ventricle; RA: Right Atrium

Due to limited transthoracic echocardiographic windows, a TEE was performed. LV function was mildly reduced, and right ventricular (RV) function was normal. However, an enlarged liver was noted to be abutting the RV and compressing the right atrium (RA), consistent with extracardiac tamponade (Figures 3-4). Mitral early diastolic wave velocities had 43% respirophasic variation, and there was end-diastolic collapse of the RA, as well as extrinsic compression at the inferior vena cava/RA junction (Figures 3-4).

The patient was stabilized after being transfused with 5 units of packed red blood cells, 4 units of fresh frozen plasma, 10 pooled units of cryoprecipitate, and 1 unit of platelets. CT confirmed an American Association for the Surgery of Trauma (AAST) grade V liver injury involving all hepatic lobes, capsular disruption, and hemoperitoneum. A multidisciplinary decision was made to pursue conservative management. The patient died following withdrawal of care.

## Discussion

These cases highlight the spectrum of intra-abdominal complications following CPR and underscore the importance of maintaining a broad differential diagnosis in patients with hemodynamic instability after ROSC. While musculoskeletal injuries such as rib and sternal fractures are the most commonly reported complications, solid organ injuries, including hepatic and splenic trauma, are increasingly recognized and may contribute significantly to morbidity [3-5,8]. Furthermore, many post-cardiac arrest patients, particularly those with suspected acute coronary syndromes, receive anticoagulation and dual antiplatelet therapy as part of standard management. These therapies, while often necessary, may exacerbate bleeding from occult CPR-related visceral injuries and contribute to progressive hemorrhagic shock when these injuries are not recognized early. In the first case, the administration of intravenous heparin, ticagrelor, and tenecteplase for STEMI management likely increased the severity of hemorrhage following CPR-related splenic injury.

The incidence of resuscitation-related injuries is substantial, with studies showing that advanced imaging modalities, such as head-to-pelvis CT, can identify a high prevalence of traumatic findings following cardiac arrest. Despite this, current post-resuscitation care primarily focuses on neurologic evaluation, cardiac assessment, and coronary reperfusion strategies, with less emphasis on detecting intra-abdominal complications [1,2,5,8].

POCUS, particularly the eFAST, plays a critical role in the early evaluation of unstable patients by clinicians at the bedside. It provides a rapid, bedside method to detect hemoperitoneum, pneumothorax, hemothorax, and pericardial effusion. In the first case, early identification of intra-abdominal hemorrhage using POCUS prompted timely surgical intervention, resulting in a favorable outcome. This supports the integration of routine bedside ultrasonography in the evaluation of post-resuscitation shock when the etiology is unclear [7,9].

The second case demonstrates a rare but clinically significant phenomenon: extracardiac tamponade. Unlike classic cardiac tamponade, which is caused by pericardial fluid accumulation, extracardiac tamponade results from external compression of the cardiac chambers. The patient in the second case became hemodynamically unstable following ROSC, and our initial differential diagnosis included myocardial stunning, recurrent myocardial ischemia, RV failure, and classic pericardial tamponade. Because these diagnoses are encountered more frequently than traumatic intra-abdominal injuries, intra-abdominal hemorrhage was not initially suspected. A progressive decline in the patient's hemoglobin concentration prompted consideration of an alternative etiology for the persistent shock. The traditional FAST protocol evaluates four regions: the pericardium (to detect cardiac tamponade), the right upper abdominal quadrant, the left upper abdominal quadrant, and the pelvis. The eFAST protocol expands this examination by including an assessment of the pleural spaces for hemothorax and pneumothorax [9].

In this case, several imaging findings favored extracardiac tamponade over conventional pericardial tamponade. TEE revealed a large hepatic hematoma causing marked external compression of the RA and RV, leading to impaired diastolic filling and hemodynamic compromise. Although only a trivial pericardial effusion was present, TEE also demonstrated significant respirophasic variation in mitral inflow velocities and compression at the inferior vena cava-right atrial junction. These findings, together with pulmonary artery catheter hemodynamics demonstrating elevation and equalization of filling pressures, supported a diagnosis of extracardiac tamponade physiology (Table 2). The absence of a significant pericardial effusion made the diagnosis particularly challenging and highlighted an important limitation of conventional diagnostic approaches [3,4].

Transthoracic echocardiography may be insufficient in critically ill patients because of poor acoustic windows. In contrast, TEE provides superior visualization of cardiac structures and allows for the identification of alternative causes of cardiac compression. Rescue TEE has emerged as a valuable bedside tool and, in this case, was essential in confirming the diagnosis of extracardiac tamponade and guiding clinical decision-making [8].

There should be a multimodal imaging approach in the evaluation of post-resuscitation shock. While CT remains the gold standard for identifying intra-abdominal injury, it may not be feasible in hemodynamically unstable patients or adequately capture dynamic physiologic processes, such as cardiac compression. These cases are also particularly relevant to anesthesiologists and intensivists who frequently manage post-cardiac arrest patients and routinely employ perioperative echocardiography and POCUS. Integrating POCUS and TEE enables rapid identification of both intra-abdominal hemorrhage and atypical causes of hemodynamic instability [1,2,6,9].

## Conclusions

Solid organ injury is an important and under-recognized complication of CPR that may contribute to post-resuscitation hemodynamic instability. POCUS and TEE provide complementary diagnostic value for identifying both intra-abdominal hemorrhage and causes of cardiac dysfunction. This case report highlights the need to expand the diagnostic framework of post-resuscitation shock to include intra-abdominal hemorrhage and extracardiac cardiac compression, particularly in patients with unexplained hemodynamic instability.
